# Characterization of LrgAB as a stationary phase-specific pyruvate uptake system in *Streptococcus mutans*

**DOI:** 10.1186/s12866-019-1600-x

**Published:** 2019-10-12

**Authors:** Sang-Joon Ahn, Kamal Deep, Matthew E. Turner, Ivan Ishkov, Anthony Waters, Stephen J. Hagen, Kelly C. Rice

**Affiliations:** 1 Department of Oral Biology, College of Dentistry, University of Florida, P.O. Box 100424, Gainesville, FL 32610 USA; 2 Department of Microbiology and Cell Science, Institute of Food and Agricultural Sciences, University of Florida, Gainesville, FL 32611 USA; 3 Department of Physics, College of Liberal Arts and Sciences, University of Florida, Gainesville, FL 32611 USA

**Keywords:** *Streptococcus mutans*, Oxidative stress, Pyruvate, Glucose metabolism, LrgAB

## Abstract

**Background:**

Our recent ‘-omics’ comparisons of *Streptococcus mutans* wild-type and *lrgAB-*mutant revealed that this organism undergoes dynamic cellular changes in the face of multiple exogenous stresses, consequently affecting its comprehensive virulence traits. In this current study, we further demonstrate that LrgAB functions as a *S. mutans* pyruvate uptake system.

**Results:**

*S. mutans* excretes pyruvate during growth as an overflow metabolite, and appears to uptake this excreted pyruvate via LrgAB once the primary carbon source is exhausted. This utilization of excreted pyruvate was tightly regulated by glucose levels and stationary growth phase *lrgAB* induction. The degree of *lrgAB* induction was reduced by high extracellular levels of pyruvate, suggesting that *lrgAB* induction is subject to negative feedback regulation, likely through the LytST TCS, which is required for expression of *lrgAB*. Stationary phase *lrgAB* induction was efficiently inhibited by low concentrations of 3FP, a toxic pyruvate analogue, without affecting cell growth, suggesting that accumulated pyruvate is sensed either directly or indirectly by LytS, subsequently triggering *lrgAB* expression. *S. mutans* growth was inhibited by high concentrations of 3FP, implying that pyruvate uptake is necessary for *S. mutans* exponential phase growth and occurs in a Lrg-independent manner. Finally, we found that stationary phase *lrgAB* induction is modulated by hydrogen peroxide (H_2_O_2_) and by co-cultivation with H_2_O_2_-producing *S. gordonii*.

**Conclusions:**

Pyruvate may provide *S. mutans* with an alternative carbon source under limited growth conditions, as well as serving as a buffer against exogenous oxidative stress_._ Given the hypothesized role of LrgAB in cell death and lysis, these data also provide an important basis for how these processes are functionally and mechanically connected to key metabolic pathways such as pyruvate metabolism.

## Background

Development of a mature biofilm on the tooth surface is the central event in the pathogenesis of dental caries [1]. This process primarily requires that cariogenic organisms, including *Streptococcus mutans*, withstand the limited resources or environmental fluctuations experienced in the oral cavity [2, 3]. An emerging concept for biofilm maturation is that the survival and persistence of these organisms during biofilm development may be mediated by regulated cell death and lysis processes, consequently eliminating bacterial cells damaged by adverse environments and benefiting the rest of the population within the biofilm [4, 5]. Toward this end, we have studied the *S. mutans* Cid/Lrg system, consisting of two dicistronic operons *lrgAB* (SMU.575c/574c) and *cidAB* (SMU.1701c/1700c) [6–11]. These operons are currently annotated as encoding holin- and antiholin-like proteins. The primary basis for these annotations came from the predicted structural similarities between CidA/LrgA and the bacteriophage-encoded holin family of proteins [4–6]. Bacteriophage holins are small membrane proteins, regulating the timing and lysis of the host cell during lytic infection with inhibitor holins (antiholins) [12]. Further support was provided by previously-observed phenotypes of *Staphylococcus aureus cid* and *lrg* mutants [13–16]. Notably, in *S. mutans*, the Cid/Lrg system is also involved in comprehensive virulence traits, including antibiotic resistance, autolysis, biofilm development, genetic competence, oxidative and heat stress responses [6, 7, 9, 11], all of which are essential for successful colonization and persistence in the oral cavity. Nevertheless, the molecular details of how Cid and Lrg function to control cell death and lysis have not yet been completely elucidated in *S. mutans* and *S. aureus*. Direct evidence of their specific cellular functionality is still scarce.

A hallmark finding for the *S. mutans* Cid/Lrg system is that expression levels of *lrg* and *cid* are counterbalanced throughout the growth cycle and in response to the availability of oxygen and glucose [6, 8]. In fact, the *lrg* and *cid* operons were originally identified to be up- and down-regulated, respectively, in aerobically grown cells [17]. The response of *lrg* and *cid* to glucose levels is particularly remarkable. The *lrg* genes are highly induced in cultures containing lower levels of glucose (≤ 15 mM) but almost completely repressed in cultures containing glucose at concentrations of 20 mM and higher [6]. In contrast, *cid* expression is negligible when cells are cultured in the presence of lower glucose concentrations (≤20 mM) but increases at higher glucose concentrations (> 20 mM) [6]. Our recent study further demonstrated that CcpA is a direct regulator of expression of *cid* and *lrg* [10], and *lrgAB* expression is also governed by the LytST two-component regulatory system (TCS), located immediately upstream of the *lrgAB* genes [6, 8]. These data suggest a functional linkage between the Cid/Lrg system and metabolic pathways. This idea was also reinforced by our recent omics studies of *S. mutans* wild-type and isogenic *ΔlrgAB* mutant strains, using RNA-seq and label-free quantitative mass spectrometry, showing that a large number of genes and/or proteins involved in carbohydrate metabolism, ABC transporters, and oxidative stress adaptation were significantly altered in the *lrgAB* mutant under stress inducing culture conditions (aerobic, heat and vancomycin treatment) [7, 11].

The fact that the *lrgAB* promoter is highly active only in cells entering stationary phase, but not in cells growing exponentially, in the low-glucose condition [6], implies that LrgAB may be important for survival of cells when exogenous carbohydrate has been depleted. This *lrgAB* induction pattern was also shown in our previous microarray data, comparing RNA expression profiles of wild-type and *lytS*-deficient strains between early- and late-exponential growth phases in BHI medium [8]. When this expression data was reconstituted for comparison of early- vs. late-exponential growth phases in the wild type, it was shown that *lrgAB* (SMU.575c-574c) was dramatically upregulated (about > 900-fold) at late-exponential phase, compared to early-exponential growth phase [10]. A four-gene operon (SMU.1421 to SMU.1424), encoding the components of the pyruvate dehydrogenase complex (PDH), was also remarkably upregulated (by > 284-fold) at late-exponential phase, compared to that of early-exponential phase [10], suggesting that LrgAB may be related to pyruvate metabolism. Coincidentally, PftAB (YsbAB), homologous to LrgAB, was recently reported to function as a pyruvate transporter in *Bacillus subtilis* [18, 19]. Similarly to the observations for the *lrgAB* genes in *S. mutans* [6, 9], expression of the *ysbAB* genes was regulated by LytSR/LytST, located upstream of *ysbAB*, as well as by CcpA [20, 21]. Expression of *ysbAB* was also maximal at stationary phase of growth [18, 19] and was reduced by glucose addition [20, 21]. *Esherichia coli* also has two TCS (BtsSR and YpdAB) homologous to LytST that have been shown to regulate expression of pyruvate transporters in response to extracellular pyruvate [22–24]. Interestingly, these TCS and BtsT (high-affinity pyruvate transporter regulated by BtsSR and YpdAB) have also been recently implicated in the ability of pyruvate to rescue *E. coli* from the viable but non-culturable (VBNC) state [24]. Collectively these studies, combined with the functional and genetic similarities of LrgAB to YsbAB in *B. subtilis*, suggest that LrgAB may also function as a pyruvate transporter in *S. mutans*.

To date little is known about the role and regulation of pyruvate in *S. mutans*, as well as other oral streptococci. Pyruvate is the final product of glycolysis, as well as a major substrate for oxidative metabolism. Pyruvate is converted to acetyl-coenzyme (acetyl-CoA) by the Pdh complex or Pfl (pyruvate formate lyase), depending on the presence or absence of oxygen, or the limitation or excess of a preferred sugar (e.g. glucose) [25, 26]. Acetyl-CoA is subsequently converted to end-products of fermentative metabolism, such as lactate, acetate, acetoin and formate. By ultilizing these pathways, cells maintain redox balance (NAD+/NADH) and generate ATP, which promotes cell homeostasis. In this present study, we reveal that pyruvate is excreted during growth of *S. mutans* as an overflow metabolite, and is reimported into cells via LrgAB when the primary carbon source (i.e. glucose) becomes exhausted. We performed a series of pyruvate quantification and *lrg* promoter reporter assays in order to characterize the role and regulation LrgAB as a pyruvate uptake system. These experiments demonstrate that LrgAB expression and activity is tightly regulated at the transcriptional level and modulated by both external and internal metabolic conditions. We also show that due to the H_2_O_2_- scavenging activity of pyruvate, re-uptake of pyruvate by *S. mutans* may be influenced by interactions with H_2_O_2_-producing oral commensals, such as *S. gordonii*. Given the possible involvement of LrgAB in inducing cell death and lysis in a programed manner, the presented data provide a new basis for how cell death and lysis mechanisms in *S. mutans* may be functionally and mechanically connected to pyruvate, a metabolic signal which may modulate homeostasis and virulence of this organism.

## Methods

### Bacterial strains, plasmids, and growth conditions

*Streptococcus mutans* UA159 and its previously-constructed mutant derivatives [6, 9] were cultured in brain heart infusion (BHI) medium (Difco Laboratories, Detroit, MI) or chemically defined medium FMC [27] containing 11 mM (or 45 mM) glucose. The medium was supplemented by sodium pyruvate (Fisher Scientific), β-fluoropyruvic acid sodium salt monohydrate (3FP, Sigma-Aldrich), pyruvic acid (Sigma-Aldrich), carbonyl cyanide m-chlorophenyl hydrazine (CCCP, Sigma-Aldrich) and 2,4-dinitrophenol (DNP, Sigma-Aldrich) or hydrogen peroxide (H_2_O_2_, Fisher Scientific), as necessary. Antibiotics were used to supplement growth media in the following concentrations: spectinomycin (1 mg/ml), kanamycin (1 mg/ml), and erythromycin (10 μg/ml). Unless otherwise noted, cultures were grown at 37 °C in a 5% CO_2_, aerobic atmosphere. For aerobic growh, cultures were grown in an aerobic incubator. To achieve anaerobic conditions, sterile mineral oil was placed on top of the cultures [6, 17, 28]. For growth measurements, fresh medium was inoculated with 1 : 100 dilutions of overnight cultures of *S. mutans*. The optical density at 600 nm (OD _600_) was measured at 37 °C at 30 min-intervals using a Bioscreen C growth curve analysis system.

### Sequence analysis and alignments

Amino acid sequences for *S. mutans* UA159 LrgA/B (SMU.574/575c), *B. subtilis* 168 YsbA/B (BSU_28900/28910), *S. aureus* MRSA252 LrgA/B (SAR_0259/0260), *S. gordonii* DL1 LrgA/B (SGO_1268/1269), and *E. coli* K12 LrgA/B (YohJ/K, JW2129/2130) were retrieved from the National Center of Biotechnology Information (NCBI) Protein database or from Uniprot (www.uniprot.org). Alignments were then generated utilizing the T-COFFEE M-coffee protein alignment tool [29] with shading completed via BoxShade version 3.21 (https://embnet.vital-it.ch/software/BOX_form.html). Percent identity matrices were then produced using the same sequences using Clustal Omega [30].

### Microplate reporter assay

GFP activities of the *S. mutans* strains harboring P*lrg-gfp* gene fusion, previously constructed [10], were observed using a Synergy microplate reader (BioTek) controlled by Gen5 software [10, 31, 32]. Overnight cultures were diluted 1:50 into 2 ml of FMC medium and grown to an OD_600_ = 0.5. At this point, these cultures were diluted 1:50 into 175 μl FMC in individual wells of a 96-well plate (black walls, clear bottoms; Corning). To evaluate the possible role of pyruvate as an antioxidant against H_2_O_2_ in the oral cavity, the reporter strain was also cultivated with *Streptococcus gordonii* DL1, a H_2_O_2_-producing oral commensal, at a ratio of 1:1. The optical density at 600 nm (OD_600_) and green fluorescence were monitored (sensitivity = 45; excitation = 485 nm; emission = 520 nm) at 30 min intervals. The fluorescence of wild-type harboring plasmid without the reporter gene fusion was subtracted from fluorescence readings of the *S. mutans* strains harboring P*lrg-gfp* gene fusion. The results are representative of at least three independent replicates, each performed in triplicate.

### Measurement of extracellular pyruvate and glucose levels

*S. mutans* UA159 wild-type and isogenic mutants (Δ*lrgAB*, Δ*lytS*, Δ*cidB*, or UA159/184*-cidAB*) strains were grown in chemically defined FMC medium, supplemented with either 11 mM or 45 mM glucose. For time course measurements of extracellular pyruvate during growth, samples (200 μl) were taken at 1–2 h intervals and half of this volume (100 μl) was used to measure the OD_600_ in a spectrophotometer for monitoring growth. The other half (100 μl) was centrifuged for 2 min at 18,000 xg to remove the cells, and pyruvate and glucose concentrations of the supernatant were quantified with an EnzyChrom™ pyruvate assay kit (BioAssay Systems, Hayward, CA) or glucose (HK) assay kit (Sigma-Aldrich), respectively, according to the manufacturer’s instructions. The results are average or representative of at least two independent replicates, each performed in duplicate.

## Results

### Sequence analysis of LrgA and LrgB homologues

**Fig. 1:**
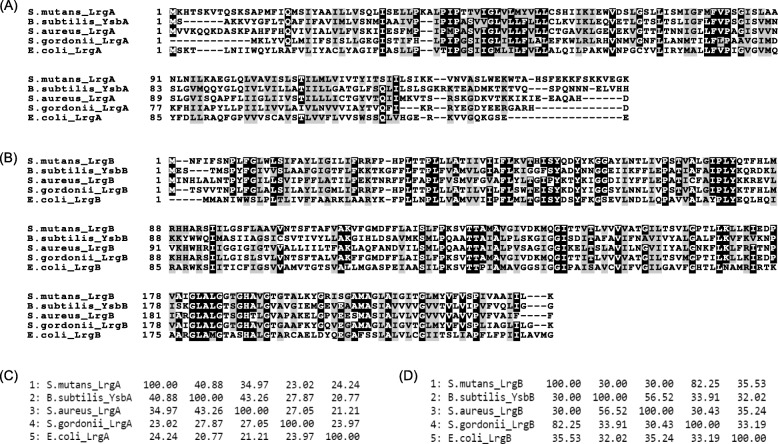
Sequence comparison of LrgA and LrgB homologues in various model bacteria (*S. mutans*, *B. subtilis*, *S. aureus*, *S. gordonii*, and *E. coli).* The amino acid sequence alignment data of LrgA (**a**) or LrgB (**b**) homologues were collected via the MCoffee program (http://tcoffee.crg.cat/apps/tcoffee/do:mcoffee) within TCoffee (http://tcoffee.crg.cat/), and the output data were printed by BoxShade (https://embnet.vital-it.ch/software/BOX_form.html). By using Clustal Omega (http://www.clustal.org/omega/), calculating the similarity between every pair of sequences in a given data set without requiring pre-alignment of the data, the similarity matrices were gererated for the amino acid sequences of LrgA (**c**) or LrgB (**d**) homologues

To evaluate the possibility that *S. mutans* LrgAB functions as a pyruvate transporter, we performed multiple sequence alignments alongside *B. subtilis* YsbA and YsbB, recently reported to be putative pyruvate transporters [18, 19], as well as other LrgA and LrgB homologues from *S. aureus*, *E. coli* and *S. gordonii* (a non-cariogenic inhabitant of the oral cavity). In the alignment of LrgA homogues (Fig. 1a), the central portion of the proteins displayed many conserved residues while N- and C-teminal portions were quite variable. The size of the other LrgA homologues was relatively shorter than that of *S. mutans* LrgA. In contrast, LrgB homologues had a similar size and displayed more conserved residues throughout the sequence (Fig. 1b). We also generated percent identity matrices using the amino acid sequences of LrgA or LrgB homologues via Clustal Omega. As shown in the comparison look-up tables for LrgA (Fig. 1c) and LrgB (Fig. 1d) homologues, each pair-wise comparison displayed an identify value greater than 20%. In particular, *S. mutans* LrgA and LrgB proteins displayed high identify to *B. subtilis* YsbA (40.9%) and YsbB (30.0%), as well as to *S. aureus* LrgA (34.9%) and LrgB (30.0%), proposed to be involved in the regulation of cell death [4, 13–15]. The YsbA and YsbB proteins also displayed higher similarities (43.3 and 56.5%) to *S. aureus* LrgA and LrgB. It is also notable that *S. mutans lytST*, known to regulate *lrgAB* [6, 8], has a similar genomic organization to these operons in *B. subtilis* and *S. aureus* (Additional file 1: Figure S1). This conservation of genomic organization and amino acid similarities suggest that *S. mutans* LrgAB may function as a pyruvate transporter, as previously observed for YsbAB in *B. subtilis* [18, 19]*.*

### *lrgAB* encodes a pyruvate uptake system in *S. mutans*

**Fig. 2:**
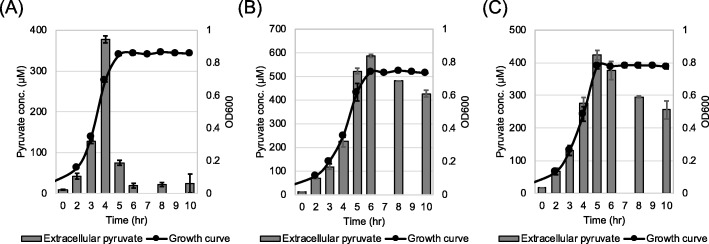
Measurement of extracellular pyruvate during growth of *S. mutans* wild-type, Δ*lrgAB*, and Δ*lytS* strains in low-glucose media. The strains, including wild type UA159 (**a**), Δ*lrgAB* (**b**), and Δ*lytS* (**c**) strains, were grown in a chemically defined medium (FMC) supplemented by 11 mM glucose. For time course measurements of extracellular pyruvate and growth, samples were taken at 1 or 2 h intervals (see Materials and Methods for details). The concentration of pyruvate was determined using an EnzyChrom™ pyruvate assay kit, and growth was measured by the optical density at 600 nm (OD_600_). Bars indicates the average concentration of extracellular pyruvate; solid line with circles indicates the corresponding growth curve. The results are average of two independent experiments. Error bars = standard deviation

Pyruvate is excreted as an overflow metabolite and re-consumed after other favorable carbon sources (i.e. glucose) are exhausted [33]. Many bacterial species, including *B. subtilis* [18, 19] and *E. coli* [22, 23] are able to utilize extracellular pyruvate as a sole carbon source, but this does not appear to be the case for *S. mutans*, as it is not able to grow in chemically defined FMC medium containing pyruvate as the sole carbon source (Additional file 2: Figure S2). Therefore, to determine whether LrgAB is involved in pyruvate transport, we first monitored extracellular pyruvate levels throughout planktonic culture growth of wild-type and Δ*lrgAB* mutant strains using a pyruvate assay kit. For this, we cultivated both strains in FMC medium, containing a low level of glucose (11 mM), allowing a strong stationary phase induction of *lrgAB* [6], at 5% CO_2_ atmosphere. As shown in Fig. 2a, the wild-type cells excreted pyruvate into the extracellular medium in a growth-phase dependent manner until late-exponential phase, at which point extracellular pyruvate concentration reached its maximum level (≈ 400 μM). Shortly afterwards, pyruvate concentrations rapidly dropped almost to zero within a hour after the onset of stationary phase (Fig. 2a). In contrast, in the stationary phase of Δ*lrgAB* cultures, the level of excreted pyruvate was only slightly decreased without dramatic change (Fig. 2b), suggesting that LrgAB may be responsible for uptake and further utilization of excreted pyruvate at stationary phase. However, the lack of LrgAB could not completely block pyruvate uptake (Fig. 2b), suggesting the presence of an additional pyruvate uptake system, likely in a Lrg-independent manner, in *S. mutans*. It is notable that pyruvate excretion throughout growth was not abolished in the Δ*lrgAB* strain, indicating that LrgAB is not likely involved in pyruvate export. The maximum level (approx. 600 μM) of pyruvate excreted during growth of the Δ*lrgAB* strain was about 50% elevated compared to wild type, and was reached after a delay to the onset of stationary phase, suggesting that lack of LrgAB may slow down pyruvate metabolism, consequently increasing the overflow of pyruvate. This is consistent with our recent RNA-seq data, showing that many metabolic pathway-related genes, including *pfl*, were significantly downregulated during growth of the Δ*lrgAB* strain [11]. Inhibition of stationary phase pyruvate uptake was also observed in the Δ*lytS* strain (Fig. 2c), indicating that the function of LrgAB as a pyruvate uptake system, is under the tight control of *lytST*, located immediately upstream of *lrgAB* [6, 8]. Given the potential interaction between *lrg* and *cid* [6, 9], we also tested whether Cid could contribute to pyruvate uptake or utilization. To test this, we quantified extracellular pyruvate during growth of Δ*cidB*, previously shown to have a similar transcriptomic profile and phenotypes as those observed for the Δ*lrgAB* mutant, particularly in response to oxidative stress [6, 9, 11]. However, the Δ*cidB* strain behaved like wildtype in terms of pyruvate excretion and uptake (Additional file 3: Figure S3). Even in the cultures of strains lacking (Δ*cidAB*) or overexpressing *cidAB* (UA159/184-*cidAB*) [9]*,* no obvious difference was observed (data not shown). Thus, we concluded that the Cid proteins are not likely involved in pyruvate uptake/utilization in *S. mutans*, thus no further investigation was performed on the *cid* operon in this study.

### Glucose and oxygen levels are important for stationary-phase pyruvate uptake of *S. mutans*

**Fig. 3:**
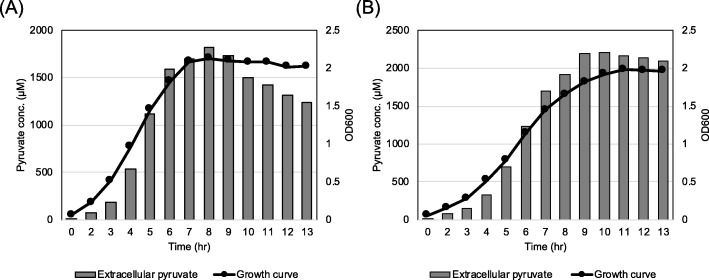
Measurement of extracellular pyruvate during growth of *S. mutans* wild-type and Δ*lrgAB* strains in high-glucose media. The wild type UA159 (**a**) and Δ*lrgAB* (**b**) strains were grown in FMC medium supplemented by 45 mM glucose. For time course measurements of extracellular pyruvate and growth, samples were taken at 1 or 2 h intervals (see Materials and Methods for details). The concentration of pyruvate was determined using an EnzyChrom™ pyruvate assay kit, and growth was measured by the optical density at 600 nm (OD_600_). Bars indicate the concentration of extracellular pyruvate; solid line with circles indicates growth curve. The results are representative of two independent experiments

We previously described that stationary phase expression of *lrgAB* was dramatically repressed in cultures containing glucose at concentrations of 20 mM and higher, although still detectable at concentrations of up to 45 mM [6]. In accordance with this observation, stationary phase pyruvate uptake was profoundly inhibited, although pyruvate was excreted at much higher levels relative to low glucose cultures (Fig. 2a), when the wild-type strain was cultivated in FMC medium containing 45 mM glucose (Fig. 3a). This inhibition by high-glucose levels was even more pronounced in the Δ*lrgAB* culture (Fig. 3b), compared to that observed for the low-glucose culture of this mutant (Fig. 2b). These results suggest that LrgAB activity, as a pyruvate uptake system, is strongly repressed in high-glucose cultures, a phenotype which is regulated at the transcriptional level [6, 10]. We additionally measured the concentration of extracellular glucose in the same time-course samples taken for the above pyruvate assays, and observed that most of glucose was rapidly depleted during exponential growth in both wild type and Δ*lrgAB* cultures, even in the glucose-excess (45 mM) condition (Additional file 4: Figure S4), suggesting that the effect of LrgAB on pyruvate uptake is not a result of altered glucose depletion. Nevetheless, pyruvate accumulation during growth was moderately reduced at stationary phase (Fig. 3a), further supporting the idea that uptake of pyruvate can also occur in a LrgAB-independent manner, because expression of *lrgAB* is almost completely inhibited at stationary phase [10].

*S. mutans* metabolizes a large proportion of glucose only as far as pyruvate and acetyl CoA, and their subsequent metabolism depends on the availability of oxygen, particularly when the glucose concentration is limited [26, 34, 35]. Our previous data showed that expression of *lrgAB* was highly upregulated in response to oxygen, and its deficiency rendered the organism super-sensitive to oxygen [6, 11, 17], suggesting that oxygen availability may influence the efficacy of LrgAB to function as a pyruvate uptake system. To test this, we quantified extracellular pyruvate in both wild type and Δ*lrgAB* mutant cells, cultivated aerobically or anaerobically. To achieve anaerobic conditions, sterile mineral oil was placed on top of the cultures. All cultures were incubated in an aerobic incubator. The overall extracellular pyruvate profile during growth of both wild type (Additional file 5: Figure S5A and S5C) and Δ*lrgAB* (Additional file 5: Figure S5B and S5D) cultures were similar between the cells, grown aerobically and anaerobically. However, we found that pyruvate was about 50% more excreted during aerobic growth (Additional file 5: Figure S5A and S5B), relative to that observed during anaerobic growth (Additional file 5: Figure S5C and S5D) in both strains, respectively. We also found that the re-uptake of excreted pyruvate was somewhat inhibited during aerobic growth in both strains, relative to anaerobic growth, suggesting that pyruvate consumption may be decelerated during aerobic growth. Overall, these results suggest that LrgAB activity may be modulated by internal and external metabolic conditions of the cell.

### Characterization of *lrgAB* expression and pyruvate uptake in response to extracellular pyruvate

**Fig. 4:**
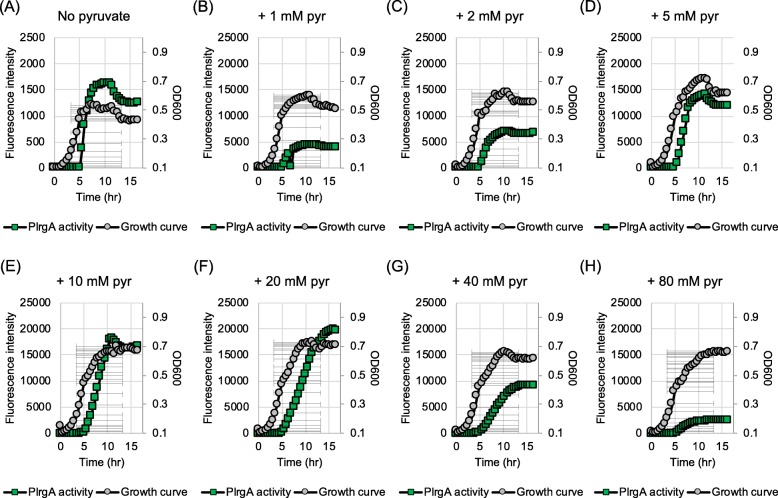
Change of *lrg* promoter (P*lrgA*) activity during growth in FMC medium supplemented by different concentrations of extracellular pyruvate. The P*lrgA-gfp* reporter strain was grown in a low-glucose (11 mM) FMC medium supplemented by 0 (**a**), 1 (**b**), 2 (**c**), 5 (**d**), 10 (**e**), 20 (**f**), 40 (**g**), and 80 mM (**h**) pyruvate (pyr). Relative *gfp* expression (green squares) and OD_600_ (grey circles; OD) were monitored during growth on a plate reader (see Materials and Methods for details). The results are representative of two independent experiments

**Fig. 5:**
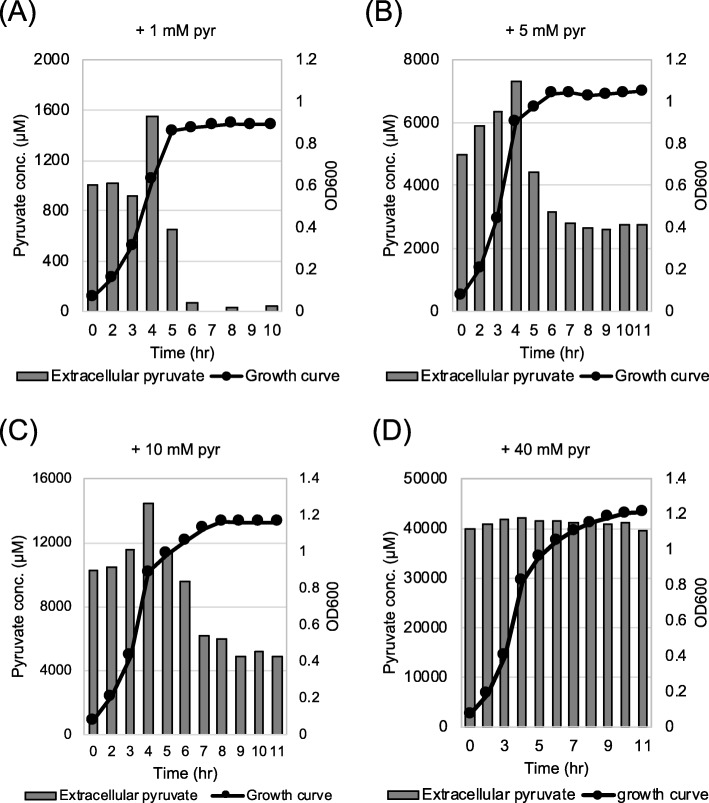
Measurement of extracellular pyruvate during growth of *S. mutans* wild-type strain in FMC medium supplemented by different concentrations of extracellular pyruvate. The *S. mutans* UA159 wild type strain was grown in a low-glucose (11 mM) FMC medium supplemented by 1 (**a**), 5 (**b**), 10 (**c**), and 40 mM (**d**) pyruvate (pyr). For time course measurements of extracellular pyruvate and growth, samples were taken at 1 or 2 h intervals (see Materials and Methods for details). The concentration of pyruvate was determined using an EnzyChrom™ pyruvate assay kit, and growth was measured by the optical density at 600 nm (OD_600_). Bars indicate the concentration of extracellular pyruvate; solid line with circles indicates growth curve. The results are representative of two independent experiments

The similar regulatory trend between pyruvate uptake (Fig. 2 and 3) and *lrgAB* expression [6, 10] suggests that re-uptake of excreted pyruvate likely depends on the level of *lrgAB* expression. However, the observation that stationary phase pyruvate uptake was not triggered by glucose depletion, prompted us to test whether the level of accumulated pyruvate is the primary stimulus for *lrgA*B induction and subsequent pyruvate uptake. To assess the pyruvate concentration dependence of *lrgAB* induction, we evaluated how *lrgAB* reponds to different concentrations of external pyruvate during growth, using the P*lrgA-gfp* reporter strain from our recent study [10]. Because growth in high levels of glucose abrogates stationary phase induction of *lrgAB*, the strain was cultivated under a low-glucose (11 mM) condition, and under aerobic conditions leading to higher induction. We initially added 1 mM pyruvate into the medium at the beginning of growth, because about 400–800 μM pyruvate would be excreted in the same growth condition in the absence of pyruvate (Figs. 2, Additional files 2 and 4: Figure S2, and Figure S4). We further reasoned that supplying extracellular pyruvate in excess may lead to early induction of *lrgAB*, which is normally induced at stationary growth phase [6, 10]. Supplementation of 1 mM exogenous pyruvate at time of inoculation elevated the *lrgAB* induction level by about 3-fold, but this induction still occurred at stationary growth phase (Fig. 4b). The induction level of *lrgAB* increased linearly with increasing concentrations of pyruvate up to 10 mM (Figs. 4b-4e). The increment was somewhat alleviated at 20 mM and further increase of supplemented pyruvate (40 and 80 mM) led to a drastic decrease of *lrgAB* expression (Figs. 4f-4h). These results suggest that the magnitude of stationary phase *lrgAB* expression is modulated by levels of extracellular pyruvate but this metabolite does not alter the timing of *lrgAB* expression during growth. To evaluate whether the transcriptional response of *lrgAB* is correlated with the capacity of the organism to take up pyruvate, we monitored the level of extracellular pyruvate during growth in a low-glucose FMC medium supplemented by additional pyruvate of 1, 5, 10 and 40 mM. When the cell was cultivated in the presence of 1 mM pyruvate, the re-uptake of pyruvate normally occurred at stationary phase (Fig. 5a). Notably, however, supplying ≥5 mM pyruvate at the time of inoculation remarkably reduced the re-uptake of extracellular pyruvate with considerable amounts of pyruvate left in the media (Figs. 5b-5d). *S. mutans* cells grown at pyruvate concentrations of 10 mM or lower (Figs. 5a-5c), excreted pyruvate in a growth-dependent manner until late-exponential growth phase, but the magnitude of changes in the increment was several-fold greater than that of cells cultivated in the absence of pyruvate. For example, maximum increments (reached at 4 h) of pyruvate excreted in the pyruvate supplemented cultures (~ 600 μM in Fig. 5a, ~ 2.0 mM in Fig. 5b, and ~ 4 mM in Fig. 5c) were much higher than no pyruvate cultures (~ 400 μM in Fig. 2a), suggesting that extracellular pyruvate can stimulate excretion of intracellular pyruvate. When cells were cultivated in the presence of 40 mM pyruvate, no profound change was observed in the pyruvate flux during growth and in stationary phase (Fig. 5d), which is in accordance with reduced *lrgAB* expression in the same culture condition (Fig. 4g). Collectively, these results suggest that extracellular pyruvate triggers expression of *lrgAB*, leading to re-uptake of pyruvate by LrgAB. These data also suggest that *lrgAB* induction can be negatively regulated by high levels of extracellular pyruvate.

### The toxic pyruvate analogue 3FP interferes with the response of *lrgAB* to extracellular pyruvate

**Fig. 6:**
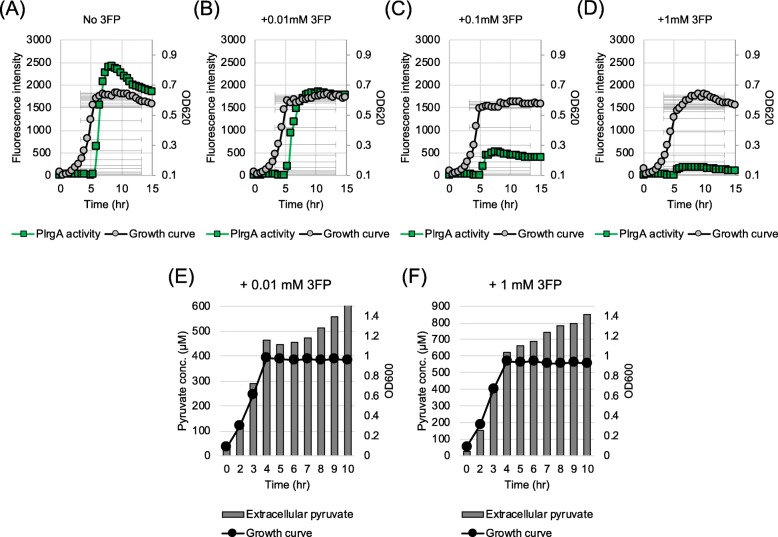
The effect of 3-fluoropyruvate (3FP; pyruvate analogue) on *lrg* promoter (P*lrgA*) activity and pyruvate uptake. A-D: Measurment of *lrg* promoter (P*lrgA*) activity. The P*lrgA-gfp* reporter strain was grown in a low-glucose (11 mM) FMC medium supplemented by 0 (**a**), 0.01 (**b**), 0.1 (**c**), or 1 mM (**d**) 3FP. Relative *gfp* expression (green square) and OD_600_ (grey circle; OD) were monitored on a plate reader (see Materials and Methods for details). The results are representative of two independent experiments. E-F: Measurement of extracellular pyruvate. The *S. mutans* UA159 wild type strain was grown in a low-glucose (11 mM) FMC medium supplemented by 0.01 (**e**) and 1 mM (**f**) 3FP. For time course measurements of extracellular pyruvate and growth, samples were taken at 1 or 2 h intervals (see Materials and Methods for details). The concentration of pyruvate was determined using an EnzyChrom™ pyruvate assay kit, and growth was measured by the optical density at 600 nm (OD_600_). Bars indicate the concentration of extracellular pyruvate; solid line with circles indicates growth curve. The results are representative of two independent experiments

It is likely that the stationary phase induction of *lrgAB* occurs through the activation of LytS in response to pyruvate, excreted during growth, because expression of *lrgAB* is under tight control of LytST [6]. Thus, to test if *lrgAB* is specifically induced in response to pyruvate, we cultivated the P*lrgA-gfp* reporter strain in low glucose media, containing non-inhibitory levels (0, 0.01, 0.1, 1 mM) of the pyruvate analog 3-fluoropyruvate (3FP), previously shown to compete with pyruvate and inhibit cell growth by binding to PDH complex in the cell [36–38]. As shown in Figs. 6a-6d, stationary phase induction of *lrgAB* was reduced in a dose-dependent manner by 3FP. Expression of *lrgAB* was obviously reduced in the presence of 0.01 mM 3FP (Fig. 6b), and expression was almost completely inhibited at 1 mM 3FP (Fig. 6d), likely due to 3FP outcompeting excreted pyruvate. This competition was more evident when cells were grown in the presence of pyruvate and 3FP (Additional file 6: Figure S6). This observation also indicates that the inhibitory effect of 3FPs can be diluted by the addition of an excess of pyruvate. Somewhat surprisingly, supplementation of 0.01 mM 3FP completely inhibited re-uptake of excreted pyruvate, and additional excretion was triggered at stationary growth phase (Fig. 6e). The same result was observed when cells were grown in the presence of 1 mM 3FP (Fig. 6f) but more pyruvate was excreted during growth, compared to the observation for the absence of 3FP, suggesting that part of 3FP was taken up into the cell in a Lrg-independent manner, likely affecting the normal utilization of pyruvate. This is further supported by the fact that *S. mutans* was unable to normally grow in the presence of 10 mM 3FP (Additional file 7: Figure S7). It seems that pyruvate is an effective overflow metabolite to induce the *lytST-lrgAB* circuit for pyuvate uptake, even though it may not be the only inducer of pyruvate uptake system.

### High concentrations of extracellular pyruvate can delay the stationary phase, regardless of LrgAB

**Fig. 7:**
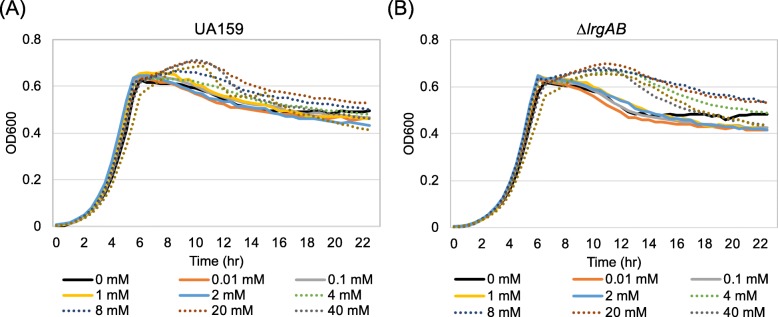
The effect of exogenously added pyruvate on the growth of *S. mutans* wild-type and Δ*lrgAB* strains. Wildtype (**a**) and Δ*lrgAB* mutant (**b**) were each grown in a low-glucose (11 mM) FMC medium supplemented by different concentrations of pyruvate (0, 0.01, 0.1, 1, 2, 4, 8, 20, 40, and 80 mM). Optical density at 600 nm was monitored every 30 min at 37 °C using the Bioscreen C lab system. The results are representative of three independent experiments

The measurements of *lrgAB* expression in the culture containing 3FP, a synthetic analogue of pyruvate, implied that pyruvate may be able to enter the cell, independently of LrgAB (Fig. 6). Increased levels of intracellular 3FP exert a negative effect on the metabolic pathways linked to the pyruvate node and eventually cell viability. We also observed prolongation of the exponential phase of growth when cells were grown in the presence of ≥40 mM pyruvate that was able to almost completely block the uptake of pyruvate (Fig. 5d). To verify this observation and further evaluate the effect of accumulated pyruvate on *S. mutans* cell growth, we monitored the growth of wild-type and Δ*lrgAB* mutant strains in FMC medium supplemented with increasing amounts of pyruvate (from 0.01 mM up to 80 mM) using a Bioscreen C plate reader. As shown in Fig. 7a, all supplemented pyruvate had no impact on the growth rate of the wildtype cells but prolonged the exponential phase of growth, delaying stationary phase, at concentrations of 4 mM or higher, which is consistent with the observation for the stationary phase uptake of pyruvate. Importantly, similar growth trends were also observed in the Δ*lrgAB* strain, showing a more evident elongation of exponential phase of growth (Fig. 7b). These results are consistent with the observation that accumulated pyruvate is taken up into the cell only at stationary phase (Fig. 2a) and inactivation of LrgAB does not completely block uptake of pyruvate (Fig. 2b). Collectively, these results further suggest the presence of another pyruvate uptake system, likely acting in a Lrg-independent manner and when concentrations of pyruvate are relatively high. The uptaken pyruvate could contribute to the survival and persistence of cells when the primary carbon source is completely consumed.

### Overflowed pyruvate can modulate the level of environmental hydrogen peroxide

**Fig. 8:**
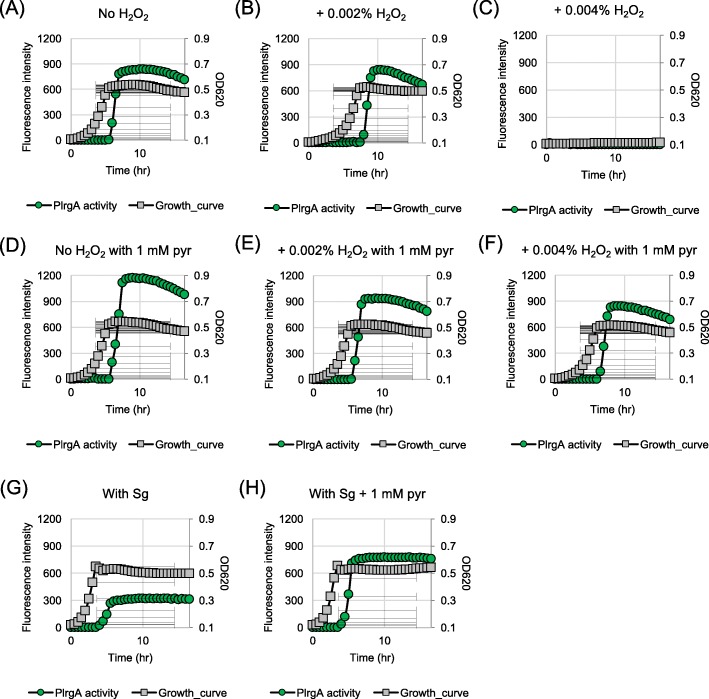
The effect of hydrogen peroxide on stationary phase *lrgAB* induction and growth. A-C: The P*lrgA-gfp* reporter strain was grown in a low-glucose (11 mM) FMC medium supplemented by 0 (**a**), 0.002 (**b**) and 0.004% (**c**) hydrogen peroxide (H_2_O_2_). D-F: The P*lrgA*-*gfp* reporter strain was grown in the same FMC medium supplemented by 0 (**d**), 0.002 (**e**) and 0.004% (**f**) hydrogen peroxide (H_2_O_2_), as well as 1 mM pyruvate (pyr). G-H: The P*lrgA*-*gfp* reporter strain was co-cultivated with *S. gordonii* DL1 (Sg) in the same FMC medium without pyruvate (**g**) and with 1 mM pyruvate (H). Relative *gfp* expression (green square) and OD_600_ (grey circle; OD) were monitored on a plate reader (see Materials and Methods for details). The results are representative of two independent experiments

It has been reported that some organic keto acids, including pyruvate, show antioxidant activity, e.g. by neutralizing oxidants such as H_2_O_2_ [39–42]. Therefore, it is possible that the induction of *lrgAB* may be influenced by interspecies interactions, especially with H_2_O_2_-producing oral commensals, including *S. gordonii*. To explore this possibility, we evaluated how *lrgAB* responds to H_2_O_2_ over growth using the P*lrgA-gfp* reporter strain, as described above. The reporter strain was cultivated in low-glucose FMC medium supplemented by 0.002% (≈0.588 mM) or 0.004% (≈1.176 mM) H_2_O_2_, and its GFP production was monitored during growth. The strain displayed an expected increased lag phase in the presence of 0.002% H_2_O_2_ (Fig. 8b) relative to that in the absence of H_2_O_2_ (Fig. 8a) but a similar stationary phase induction of *lrgAB*. At 0.004% H_2_O_2_, the strain did not grow well (Fig. 8c). Notably, the stationary phase *lrgAB* induction observed in cultures supplemented with 1 mM pyruvate and 0.002% H_2_O_2_ (Fig. 8e) was substantially reduced relative to 1 mM pyruvate alone (Fig. 8d) and without an increased lag phase (Fig. 8e), suggesting that pyruvate was used to scavenge H_2_O_2._ This H_2_O_2_-scavenging effect was more evidently observed when the strain was cultivated in the presence of 0.004% H_2_O_2,_ because the strain was able to grow normally in this condition (Fig. 8f). These results suggest that pyruvate may serve *S. mutans* as a carbon source, as well as an antioxidant against H_2_O_2,_ consequently contributing to ecological fitness of this organism in the oral cavity. To reinforce this conclusion, we cultivated the reporter strain with *S. gordonii* DL1, known to secrete substantial amounts of H_2_O_2_, in the absence and presence of 1 mM pyruvate, a concentration that has no effect on the growth of *S. mutans* (Fig. 7a) and *S. gordonii* (data not shown) but triggers *lrgAB* induction at the stationary phase. The expression of *lrgAB* was remarkably reduced by co-cultivation with *S. gordonii* (Fig. 8g) but the negative effect was almost completely complemented by exogenously adding pyruvate into the culture (Fig. 8h). These results suggest that re-uptake of accumulated pyruvate by *S. mutans* may be affected by interspecies competition, especially with H_2_O_2_-producing oral commensals, consequently altering the metabolic status of the organism .

## Discussion

The *S. mutans* Cid/Lrg system represents an excellent model to study how this organism withstands various stressors encountered in the oral cavity. Our previously-published “-omics” data suggested that the adaptation process to adverse environments requires metabolic remodeling [7–9, 11], which is in accordance with the fact that (i) *lrgAB* is specifically induced at stationary phase and (ii) is tightly regulated by glucose and oxygen levels [6, 8, 17]. The primary finding of this current study is that LrgAB is important to initiate the rapid re-uptake of pyruvate, excreted during growth, when cells confront carbon starvation/nutrient limitation in stationary growth phase. The excretion (overflow) of pyruvate is a common feature of many bacterial species when cultivated under a carbon-excess condition, contributing to metabolic balancing between carbon uptake and consumption [33, 43–45]. Because of its central role in metabolism, it is not surprising that excretion and re-uptake of pyruvate are tightly regulated at multiple levels, and the three main enzymes that utilize pyruvate as a substrate, Pdh, Pfl and Ldh, are accordingly modulated to the cell’s metabolic status and environmental condition [25, 46]. We have previously shown that the *pdh* and *pfl* genes, responsible for the conversion of pyruvate to acetyl-CoA, were highly upregulated at late-exponential phase [10], suggesting that their gene expression is linked with the uptake of pyruvate, functionally and metabolically. Given that the *pdh* genes were commonly upregulated in response to three stress conditions (aerobic, heat and vancomycin challenge) previously tested [11], pyruvate uptake and metabolism should affect the ability of *S. mutans* to adapt to suboptimal conditions. Pyruvate is likely a common nutrient in the microbiome environment, such as oral cavity. But its utilization and regulation in *S. mutans* have been poorly characterized, especially with respect to how they are connected to the virulence network of this organism.

It seems that the re-uptake of pyruvate is tightly regulated by glucose levels and stationary growth phase *lrgAB* induction. Growth in high levels of glucose abrogates stationary-phase response of *lrgAB* to extracellular pyruvate, suggesting that the response of *lrgAB* to extracellular pyruvate is subject to carbon catabolite repression. Supplementation of exogenous pyruvate was unable to induce earlier induction of *lrgAB* (normally occurring at stationary phase), reinforcing that uptake of pyruvate occurs only under limited growth, and is subject to tight metabolic control. Given that *lrgAB* expression requires activation by LytST TCS [6], repression of *lrgAB* may be primarily due to inaccessibility of LytT to the promoter region of *lrgAB*. In our recent study, we found that the *cre* site for CcpA binding overlapped, in part, with a potential LytT binding site in the promoter region of *lrgAB* [10], suggesting that activation of *lrgAB* by LytT may be prevented by CcpA binding to the promoter region of *lrgAB*. However, repression of *lrgAB* was not released even in the absence of CcpA when the cell was cultivated in high-glucose medium [10], suggesting that additional regulation(s) may be involved in the expression of *lrgAB*. In fact, CcpA often works together with CodY to sense changes in nutrient availability and coordinate, directly and indirectly, the expression of hundreds of genes involved in carbon and nitrogen metabolism of Gram-positive bacteria [45, 47–49]. CodY acts mainly as a repressor, and many genes encoding metabolic pathway components are repressed during growth in the presence of excess nutrients and involved in adaptation to poor growth conditions [50]. Accordingly, we also observed that the *codY* gene was several-fold upregulated at early-exponential phase, compared to late-exponential growth phase [8]. Thus, it is possible that these two global regulators (CcpA and CodY) interact and modulate *lrgAB* expression in response to environmental conditions and nutritional needs, which is currently under investigation.

The specific response of *lrgAB* to extracellular pyruvate was supported by the observation that supplementation of 10 μM 3FP had a profound effect on repressing the expression of *lrgAB*, likely by interfering with activation of the LytS sensor kinase by pyruvate. These results suggest that LrgAB may be responsible for facilitating the recovery from carbon starvation when concentrations of pyruvate are low. On the contrary, the finding that the stationary phase induction of *lrgAB* was alleviated by high concentrations of extracellular pyruvate (approx. > 20 mM), revealed the existence of a negative feedback regulation acting on LytST by the presence of high levels of extracellular pyruvate. This feedback regulation may contribute to balanced extracellular and intracellular pyruvate levels, although it remains to be elucidated. Given that the re-uptake of accumulated pyruvate is subject to a tight metabolic control, pyruvate may play a role as a potential metabolic signal to determine cellular fate under limited nutrient conditions. However, this regulatory model does not account for the observation that cells were still capable of utilizing extracellular pyruvate even in the absence of LrgAB, resulting in prolonged exponential growth, suggesting the existence of an additional pyruvate uptake system that operates in a LrgAB-independent manner and preferably when pyruvate concentrations are relatively high (at several mM levels). Indeed, it has been suggested that *E. coli* uses one exporter and two uptake transporters to modulate the level of intracellular pyruvate [51]. However, uptake of pyruvate does not appear to be attributed to diffusion, because the cell displayed the same growth rate even in the presence of high concentrations of pyruvate*.* It is also supported by the observation that *S. mutans* was also unable to grow in a medium containing high concentrations of pyruvate (up to 80 mM) as sole carbon source (Additional file 2: Figure S2). These multiple pyruvate uptake systems and feedback regulations may ensure a tight management of pyruvate homeostasis which in turn may facilitate cellular adaptation.

Although the data herein suggest that LrgAB is primarily responsible for uptake of pyruvate in *S. mutans*, it remains to be elucidated how LrgAB mechanistically mediates the uptake (transport) of pyruvate. In a preliminary experiment, we found that stationary phase *lrgAB* induction was significantly inhibited by two hydrophobic protonophores, CCCP (carbonyl cyanide m-chlorophenyl hydrazine) and DNP (2,4-dinitrophenol) at 1 μM and 100 μM, respectively (Additional file 8: Figure S8), suggesting that a pH gradient between interior and exterior of cell may be involved in the activation of LrgAB. Accordingly, a recent study showed that BtsT (also named YjiY) functions as a specific pyruvate/H+ symporter in E. coli [23]. This aspect is being further investigated in LrgAB. It is also noteworthy that *lrgAB* is induced by pyruvic acid, which still occurs at stationary growth phase (Additional file 9: Figure S9). Although pyruvate could freely diffuse through the lipid bilayer of the cell membrane in its protonated form [52], most of pyruvic acid should be in a dissociated, charged state (pyruvate), due to acidity of pyruvic acid (pKa = 2.5), suggesting that LrgAB may not be energized only by a proton gradient. Thus, in order to metabolize pyruvate, the uptake systems, including LrgAB, would be required for internalizing pyruvate. It also appears that other metabolic intermediates with structural similarity with pyruvate are transported by LrgAB. In our preliminary experiment using the P*lrgA-gfp* reporter strain, we observed that *lrgAB* did not repond to α-ketoglutarate, malate, oxaloacetate, and succinate, but citrate could trigger *lrgAB* induction (data not shown), suggesting that LrgAB may be specifically responsible for uptake of pyruvate. This theory is also supported by the observation that a synthetic analogue of pyruvate (3FP) effectively competed with pyruvate. This specificity also reinforces the role of pyruvate as a potential metabolic signal, because pyruvate is excreted together with other metabolic compounds. The detailed mechanism for how pyruvate is transported via LrgAB through the cell membrane is an important part of understanding the Cid/Lrg system and warrants further investigation.

Another important finding of this study was that pyruvate directly reacts with H_2_O_2_, suggesting that the re-uptake of pyruvate may be influenced by interspecies interactions, especially with H_2_O_2_-producing oral commensals, such as *S. gordonii* and *S. sanguinis* [53, 54]. The reaction of H_2_O_2_ with pyruvate is presented as CH_3_-CO-COOH+H_2_O_2_ → CH_3_-COOH+H_2_O + CO_2_ [39]. Indeed, acetate and CO_2_ are the major by-products of pyruvate reaction with H_2_O_2_, with pyruvate being produced and excreted during exponential growth while glucose is converted to biomass. Acetate would be presumably also taken up into the cell in parallel with pyruvate under nutrient limited growth condition [33, 55]. Intriguingly, the *pta-ackA* pathway which generates acetate and ATP was reported to cause cell death in *S. aureus* [56], and to be regulated by CcpA and CodY in *S. mutans* [57] and *B. subtilis* [45]. Therefore, these observations further suggest a potential connection between pyruvate metabolism and cell death, hypothesized to be induced by the Cid/Lrg system [6]. It is also noteworthy that another organic keto acid, e.g. α-keto glutarate, can effectively scavenge H_2_O_2_ [39–42], suggesting that the level of H_2_O_2_ could be modulated by multiple metabolic products. It is an on-going study how the levels of H_2_O_2_ and pyruvate (or other organic acids) are changed in the interaction between *S. mutans* and *S. gordonii* (or *S. sanguinis*), and how the LrgAB pyruvate uptake system contributes to the protection from environmental H_2_O_2_ challenge.

## Conclusions

The results of this study demonstrate that LrgAB is the first identified pyruvate uptake system in *S. mutans,* and provide an important new basis for how the hypothesized role of LrgAB in modulating cell death and lysis are mechanistically connected to key metabolic pathways. Overall, excreted pyruvate may play several important roles in *S. mutans* physiology, such as a carbon source and/or metabolic precursor for amino acid/fatty acid biosynthesis under nutrient-limited and/or stationary phase growth conditions, as well as by buffering external sources of oxidative stress, such as H_2_O_2_, which primarily appears to be modulated by LytST-LrgAB. Therefore, it is possible that the trafficking and utilization of pyruvate may significantly contribute to shaping a homeostatic mechanism and composition of oral microflora, consequently influencing the development of caries. Nevertheless, due to highly variable in vivo conditions especially in respect to nutrient availability and unknown host factors, the actual effect and role of pyruvate remain to be elucidated and are currently under investigation. This study also opens the possibility of searching for additional pyruvate uptake system as well as examining the relationship between metabolite fluxes and ecological fitness. This will be important as we explore the possible mechanisms underlying stress tolerance and programmed cell death as a bacterial survival strategy at the community level.

## Supplementary information

**Additional file 1: Figure S1.** Schematic diagram of the *lyt* and *lrg* genetic loci in the genomes of *S. mutans*, *B. subtilis*, and *S. aureus*.

**Additional file 2: Figure S2.** Growth of *S. mutans* wild type in chemically defined FMC medium containing increasing concentrations of pyruvate as the sole carbon source. Growth was monitored during growth in a Bioscreen C system that was set to shake for 15 s every 30 min. The results are representative of two independent experiments.

**Additional file 3: Figure S3.** Measurement of extracellular pyruvate during growth of *S. mutans* Δ*cidB* mutant strain in low-glucose FMC medium. The strain was grown in a chemically defined medium (FMC) supplemented by 11 mM glucose. For time course measurements of extracellular pyruvate and growth, samples were taken at 1 or 2 h intervals (see Materials and Methods for details). The concentration of pyruvate was determined using an EnzyChrom™ pyruvate assay kit, and growth was measured by the optical density at 600 nm (OD_600_). Bar indicates the concentration of extracellular pyruvate; line indicates growth curve. The results are average of two independent experiments.

**Additional file 4: Figure S4.** Measurement of extracellular glucose during growth of *S. mutans* wild type and Δ*lrgAB* strains in the high-glucose media. The strains were grown in a chemically defined medium (FMC) supplemented by 45 mM glucose. For time course measurements of extracellular pyruvate and growth, samples were taken at 1 or 2 h intervals (see Materials and Methods for details). The concentration of glucose was determined using an Glucose (HK) assay kit, and growth was measured by the optical density at 600 nm (OD_600_). Bar indicates the concentration of extracellular pyruvate; line indicates growth curve. The results are representative of two independent experiments.

**Additional file 5: Figure S5.** Measurement of extracellular pyruvate during aerobic (A and B) or anaerobic (C and D) during growth of *S. mutans* wild-type (A and C) and Δ*lrgAB* (B and D) in the low-glucose media. The strains were grown in a chemically defined medium (FMC) supplemented by 11 mM glucose. For anaerobic growth, sterile mineral oil was placed on top of cultures. For time course measurements of extracellular pyruvate and growth, samples were taken at 1 or 2 h intervals (see Materials and Methods for details). The concentration of pyruvate was determined using an EnzyChrom™ pyruvate assay kit, and growth was measured by the optical density at 600 nm (OD_600_). Bar indicates the concentration of extracellular pyruvate; line indicates growth curve. The results are average of two independent experiments.

**Additional file 6: Figure S6.** The effect of 3-fluoropyruvate (3FP; pyruvate analogue) on *lrg* promoter (P*lrgA*) activity in the presence of pyruvate. The P*lrgA-gfp* reporter strain was grown in a low-glucose (11 mM) FMC medium supplemented by 0 (A), 0.01 (B), 0.1 (C) or 1 mM (D) 3FP. Pyruvate was added into the medium at the concentration of 1 mM. Relative *gfp* expression (green square) and OD_600_ (grey circle; OD) were monitored on a plate reader (see Materials and Methods for details). The results are representative of two independent experiments.

**Additional file 7: Figure S7.** The effect of 3FP on the growth of *S. mutans* wildtype. The strain was cultivated in 11 mM glucose FMC media containing different concentrations (0, 1, 2, and 10 mM) of the pyruvate analog 3-fluoropyruvate (3FP). Growth was monitored during growth in a Bioscreen C system that was set to shake for 15 s every 30 min. The results are representative of two independent experiments.

**Additional file 8: Figure S8.** The effect of protonophores CCCP and DNP on *lrg* promoter (P*lrgA*) activity. The P*lrgA-gfp* reporter strain was grown in a low-glucose (11 mM) FMC medium (A), supplemented by 1 μM CCCP (carbonyl cyanide m-chlorophenyl hydrazine, B) and 0.1 mM DNP (2,4-dinitrophenol, C). Relative *gfp* expression (green square) and OD_600_ (grey circle; OD) were monitored on a plate reader (see Materials and Methods for details). The results are representative of two independent experiments.

**Additional file 9: Figure S9.** Change of *lrg* promoter (P*lrgA*) activity during growth in FMC medium supplemented by different concentrations of extracellular pyruvic acid. The P*lrgA-gfp* reporter strain was grown in a low-glucose (11 mM) FMC medium supplemented by 0 (A), 1 (B), and 2 mM (C) pyruvic acid (PA). Relative *gfp* expression (green squares) and OD_600_ (grey circles; OD) were monitored during growth on a plate reader (see Materials and Methods for details). The results are representative of two independent experiments.

## Data Availability

The datasets analysed during the current study are available from the corresponding author upon reasonable request.
