# Supplementary figures and images for: Characterization of LrgAB as a stationary phase-specific pyruvate uptake system in Streptococcus mutans
Source: BMC Microbiol. 2019 Oct 12;19:223. doi: 10.1186/s12866-019-1600-x (PMC6790026; doi:10.1186/s12866-019-1600-x)

**Fig. S1**

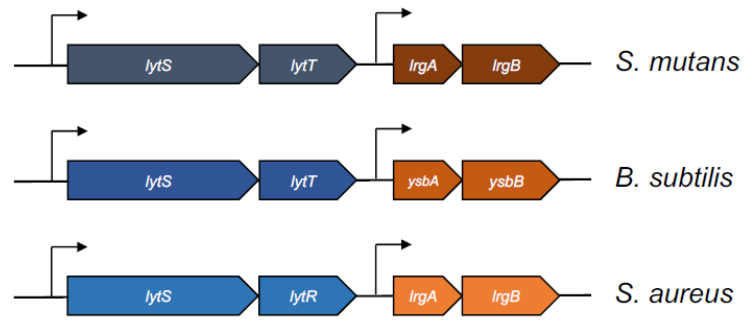

Supplement: Supplementary file 1 — Additional file 1: Figure S1. Schematic diagram of the lyt and lrg genetic loci in the genomes of S. mutans, B. subtilis, and S. aureus. [file 12866_2019_1600_MOESM1_ESM.pdf]

Fig. S2

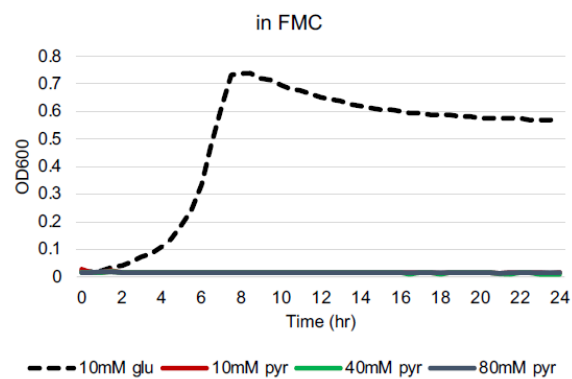

Supplement: Supplementary file 2 — Additional file 2: Figure S2. Growth of S. mutans wild type in chemically defined FMC medium containing increasing concentrations of pyruvate as the sole carbon source. Growth was monitored during growth in a Bioscreen C system that was set to shake for 15 s every 30 min. The results are representative of two independent experiments. [file 12866_2019_1600_MOESM2_ESM.pdf]

Fig. S3

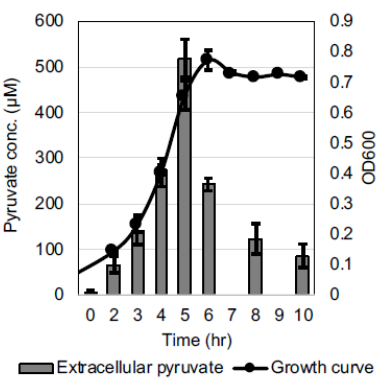

Supplement: Supplementary file 3 — Additional file 3: Figure S3. Measurement of extracellular pyruvate during growth of S. mutans ΔcidB mutant strain in low-glucose FMC medium. The strain was grown in a chemically defined medium (FMC) supplemented by 11 mM glucose. For time course measurements of extracellular pyruvate and growth, samples were taken at 1 or 2 h intervals (see Materials and Methods for details). The concentration of pyruvate was determined using an EnzyChrom™ pyruvate assay kit, and growth was measured by the optical density at 600 nm (OD600). Bar indicates the concentration of extracellular pyruvate; line indicates growth curve. The results are average of two independent experiments. [file 12866_2019_1600_MOESM3_ESM.pdf]

Fig. S4

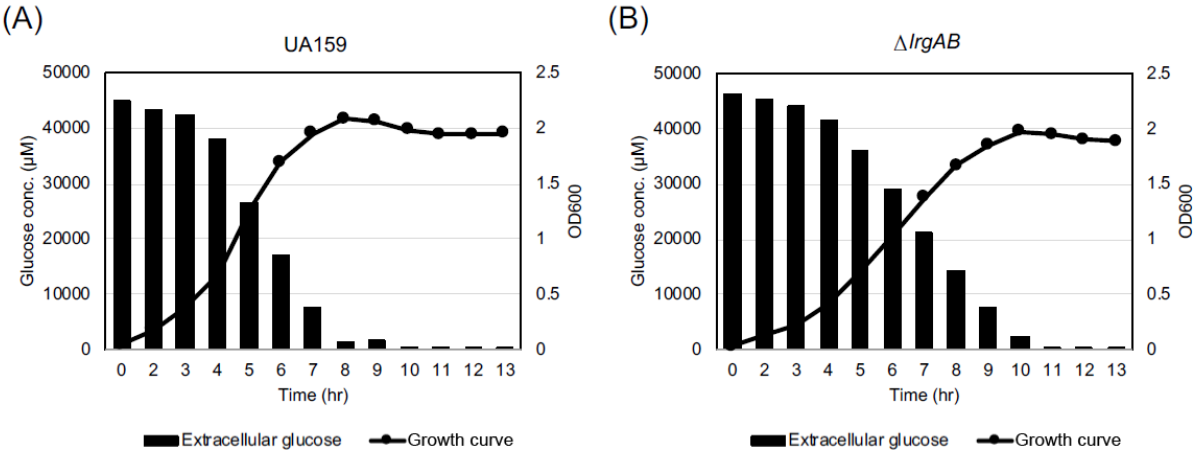

Supplement: Supplementary file 4 — Additional file 4: Figure S4. Measurement of extracellular glucose during growth of S. mutans wild type and ΔlrgAB strains in the high-glucose media. The strains were grown in a chemically defined medium (FMC) supplemented by 45 mM glucose. For time course measurements of extracellular pyruvate and growth, samples were taken at 1 or 2 h intervals (see Materials and Methods for details). The concentration of glucose was determined using an Glucose (HK) assay kit, and growth was measured by the optical density at 600 nm (OD600). Bar indicates the concentration of extracellular pyruvate; line indicates growth curve. The results are representative of two independent experiments. [file 12866_2019_1600_MOESM4_ESM.pdf]

Fig. S5

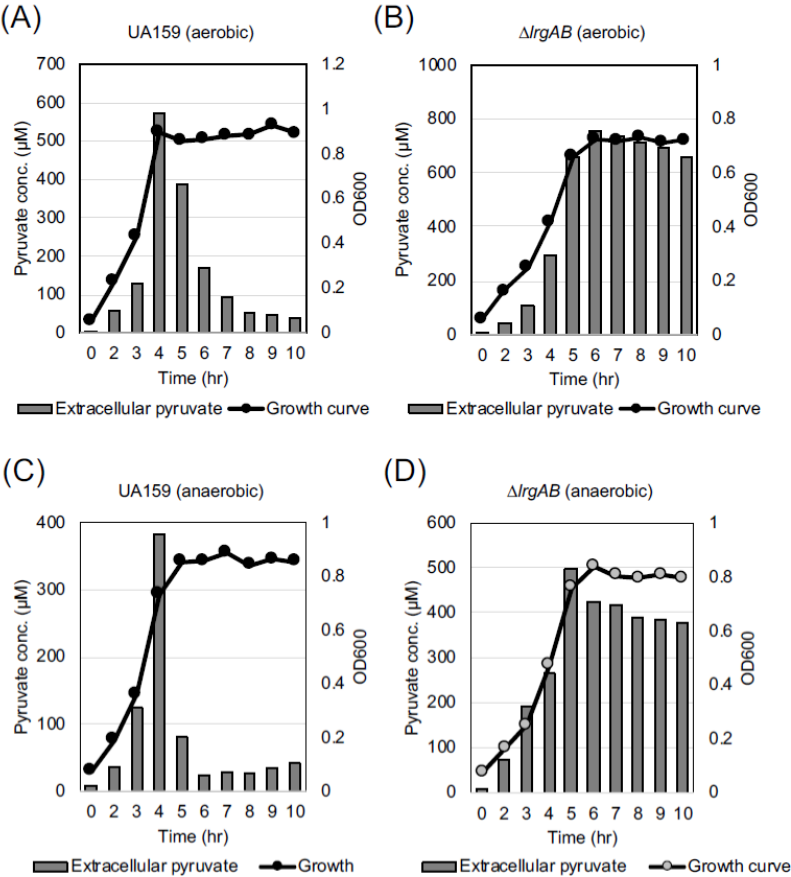

Supplement: Supplementary file 5 — Additional file 5: Figure S5. Measurement of extracellular pyruvate during aerobic (A and B) or anaerobic (C and D) during growth of S. mutans wild-type (A and C) and ΔlrgAB (B and D) in the low-glucose media. The strains were grown in a chemically defined medium (FMC) supplemented by 11 mM glucose. For anaerobic growth, sterile mineral oil was placed on top of cultures. For time course measurements of extracellular pyruvate and growth, samples were taken at 1 or 2 h intervals (see Materials and Methods for details). The concentration of pyruvate was determined using an EnzyChrom™ pyruvate assay kit, and growth was measured by the optical density at 600 nm (OD600). Bar indicates the concentration of extracellular pyruvate; line indicates growth curve. The results are average of two independent experiments. [file 12866_2019_1600_MOESM5_ESM.pdf]

Fig. S6

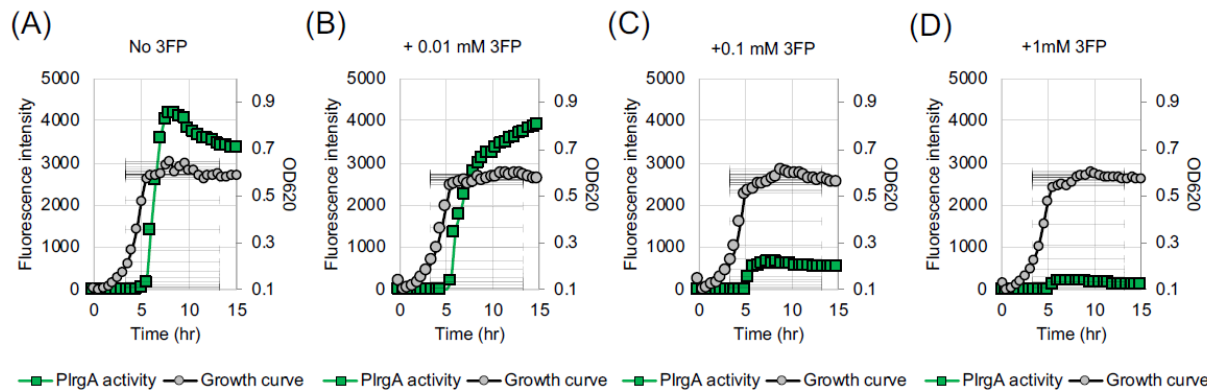

Supplement: Supplementary file 6 — Additional file 6: Figure S6. The effect of 3-fluoropyruvate (3FP; pyruvate analogue) on lrg promoter (PlrgA) activity in the presence of pyruvate. The PlrgA-gfp reporter strain was grown in a low-glucose (11 mM) FMC medium supplemented by 0 (A), 0.01 (B), 0.1 (C) or 1 mM (D) 3FP. Pyruvate was added into the medium at the concentration of 1 mM. Relative gfp expression (green square) and OD600 (grey circle; OD) were monitored on a plate reader (see Materials and Methods for details). The results are representative of two independent experiments. [file 12866_2019_1600_MOESM6_ESM.pdf]

**Fig. S7**

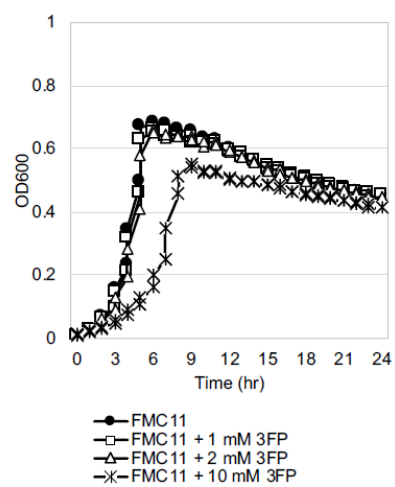

Supplement: Supplementary file 7 — Additional file 7: Figure S7. The effect of 3FP on the growth of S. mutans wildtype. The strain was cultivated in 11 mM glucose FMC media containing different concentrations (0, 1, 2, and 10 mM) of the pyruvate analog 3-fluoropyruvate (3FP). Growth was monitored during growth in a Bioscreen C system that was set to shake for 15 s every 30 min. The results are representative of two independent experiments. [file 12866_2019_1600_MOESM7_ESM.pdf]

Fig. S8

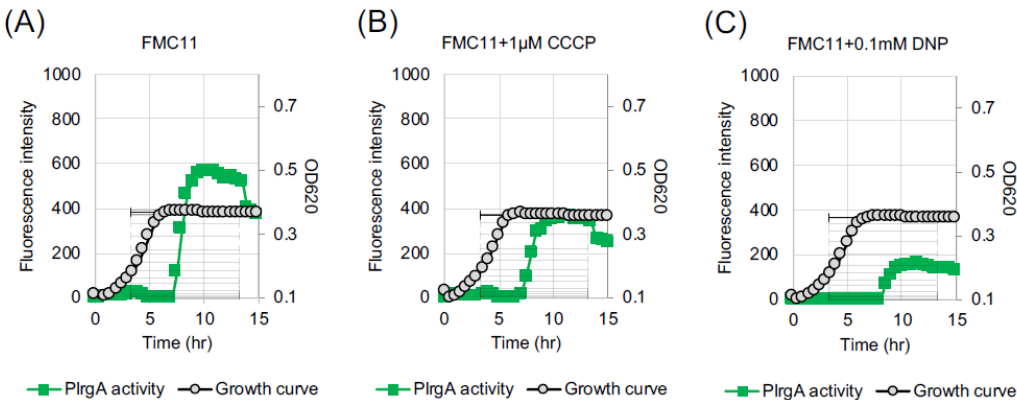

Supplement: Supplementary file 8 — Additional file 8: Figure S8. The effect of protonophores CCCP and DNP on lrg promoter (PlrgA) activity. The PlrgA-gfp reporter strain was grown in a low-glucose (11 mM) FMC medium (A), supplemented by 1 μM CCCP (carbonyl cyanide m-chlorophenyl hydrazine, B) and 0.1 mM DNP (2,4-dinitrophenol, C). Relative gfp expression (green square) and OD600 (grey circle; OD) were monitored on a plate reader (see Materials and Methods for details). The results are representative of two independent experiments. [file 12866_2019_1600_MOESM8_ESM.pdf]

Fig. S9

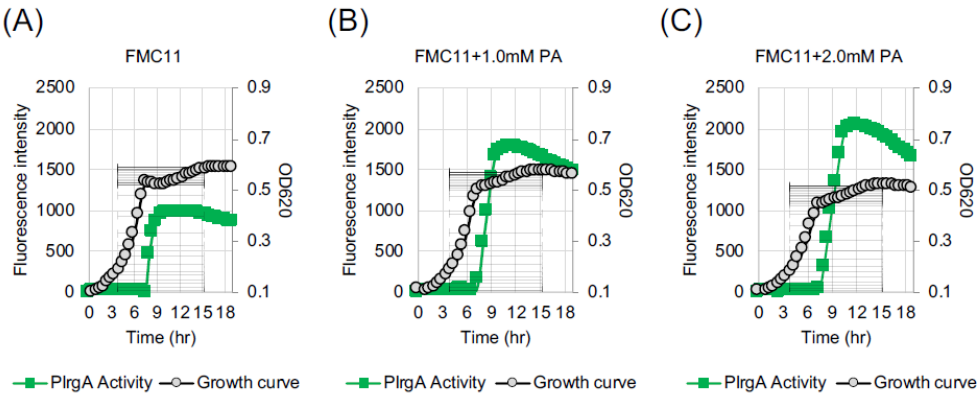

Supplement: Supplementary file 9 — Additional file 9: Figure S9. Change of lrg promoter (PlrgA) activity during growth in FMC medium supplemented by different concentrations of extracellular pyruvic acid. The PlrgA-gfp reporter strain was grown in a low-glucose (11 mM) FMC medium supplemented by 0 (A), 1 (B), and 2 mM (C) pyruvic acid (PA). Relative gfp expression (green squares) and OD600 (grey circles; OD) were monitored during growth on a plate reader (see Materials and Methods for details). The results are representative of two independent experiments. [file 12866_2019_1600_MOESM9_ESM.pdf]
